# A Review of Isolation, Chemical Properties, and Bioactivities of Polysaccharides from *Bletilla striata*

**DOI:** 10.1155/2020/5391379

**Published:** 2020-05-19

**Authors:** Xiaolong Ji, Mingsong Yin, Hui Nie, Yanqi Liu

**Affiliations:** ^1^School of Food and Bioengineering, Zhengzhou University of Light Industry, Zhengzhou 450002, China; ^2^Guangxi Talent Highland of Preservation and Deep Processing Research in Fruit and Vegetables, Hezhou University, Hezhou, China

## Abstract

Recently, polysaccharides from *Bletilla striata*, a member of the orchidaceous family, aroused the wide interest of people, especially their isolation, chemical properties, and bioactivities. It is reported that these polysaccharides are the most important biologically active components of *B. striata*, exhibiting various biological activities, such as immunomodulatory, antioxidant, antifibrotic, and hemostatic effects. This review appraised the available literatures which described different aspects of *B. striata* polysaccharides, including the extraction, separation, purification, structural characterization, and biological activities. We expect to lay the foundation for further investigation of the application of *B. striata* polysaccharides in the field of functional foods and biomedicine.

## 1. Introduction

*Bletilla striata* (Thunb.) Reichb. f. (Orchidaceae), named “Bai Ji” in Chinese, belongs to the orchidaceous family and is distributed widely in eastern Asian countries, including China, North Korea, Japan, and Burma [1, 2]. *B. striata* has been widely used in traditional Chinese herbal medicine (TCM) for thousands of years and is one of the “seven white” in ancient China, mainly distributed in the Qinling Mountains and Yangtze River region [3, 4]. TCM holds that *B. striata* is bitter, astringent, neutral, and warm and has bioactivities in alimentary canal mucosal damage, ulcers, bleeding, bruises, and burns [4, 5]. Also, the functions of hemostasis, detumescence, and improving one's health of *B. striata* were recorded by Chinese pharmacopeia (2010) [2, 6].

Increasing numbers of modern chemical and pharmacological studies revealed that *B. striata* has many beneficial effects, including healing, hemostasis, antioxidation, anti-inflammation, antifibrotic, and immunomodulatory activity *in vitro* [4, 7, 8]. These multiple pharmacological effects of *B. striata* are attributed to its various functional ingredients, such as polysaccharides, saponins, flavonoids, terpenoids, trace elements, and other chemicals [1, 4], among which the polysaccharides (BSPs) have gradually received attention and are now considered to be the main active substance of *B. striata* for its beneficial effects [9–11]. BSPs isolated using different extraction and purification methods have been confirmed to be structurally diverse biomacromolecules with various functions [2]. Their anti-inflammatory [12], antitumor [13], immunomodulatory [14], and antifibrotic [15] activities have been well recognized. Moreover, BSPs have been used as a therapeutic agent for the treatment of bleeding [16, 17]. So for, BSPs with multiple functional applications have been widely used in food and medical industries [2].

Throughout the available literatures, there has been no systematic review of *B. striata* polysaccharides. Neither has there been a systematic review on the extraction and purification techniques nor on the structural characteristics and biological activities. In this review, we intend to systematically summarize the research findings about BSPs in the past decades to provide a comprehensive insight into the extraction and purification techniques as well as the structure and pharmacological effects of BSPs.

The main purpose of this review is to provide informative knowledge which is expected to promote better utilization of these polysaccharides and to preliminarily illustrate the relationships between the structure and bioactivities of BSPs, as well as pave the way for further exploitation of these compounds in the field of biomedicine.

## 2. Extraction and Purification Methods

In the past decades, diverse methods have been described for the extraction, isolation, and purification of polysaccharides from *B. striata* [9, 18–20]. Extraction methods of BSPs from pretreated dry powders are summarized in Table 1. Generally, water extraction at a certain temperature is the classic and also most convenient method for laboratory extraction and even industrial extraction [21–23]. Extraction time and temperature have significant influences on the yield of conventional water extraction, and solid-liquid ratio is usually in the range of 1 : 5 to 1 : 50. Different extraction conditions, such as the source of *B. striata*, extraction temperature, time, solvent, and the raw material to solvent ratio, have significant effects on the extraction rate of BSPs [2, 3, 23].

Wang found the following optimum conditions for extracting BSPs using distilled water: extraction temperature—60°C; extraction time—3 h; and water : raw material ratio—10 : 1. With this method, only one round of extraction produced a final yield of 18.50% [24]. However, hot water extraction still has the disadvantages of being time consuming and having high temperature, low efficiency, and possible polysaccharide degradation.

To improve the yield of extracted BSPs, other techniques have also been applied, such as ultrasonic- [3, 25], microwave- [3, 6], and infrared-assisted [20] extraction, by promoting the breakdown of the *B. striata* cell walls [23]. For example, He et al. reported that ultrasound-assisted extraction based on an orthogonal array design could improve the extraction efficiency of polysaccharides. They also reported the following optimal extraction conditions with two rounds of extraction: ultrasonic power—400 W; extraction time—0 min; extraction temperature—60°C; and water to raw material ratio (*w* : *w*)—50 : 1. Under these conditions, the yield of crude polysaccharides from *B. striata* harvested in Shanxi province increased to 26.02% [25]. Microwave-assisted extraction has also been explored to maximize the yield of BSPs. The most favorable conditions were found to be the following: microwave power—504 W; extraction time—5 min; and water (pH 7.0) to raw material ratio (*w* : *w*)—63 : 1 [26]. In addition, with the help of infrared-assisted extraction for BSPs and the Box-Behnken design, a considerable improvement of the extraction yield has been realized. An extraction yield of 43.95 ± 0.26% was achieved using an extraction temperature of 75°C, a water to solid ratio of 53 mL/g, and an extraction time of 2.5 h [20]. In summary, we could find that each of these assisted extraction methods could contribute to shorten the processing time, reduce solvent consumption, and further decrease the economic cost while improving the extraction efficiency of BSPs [3, 6, 27].

**Figure 1:**
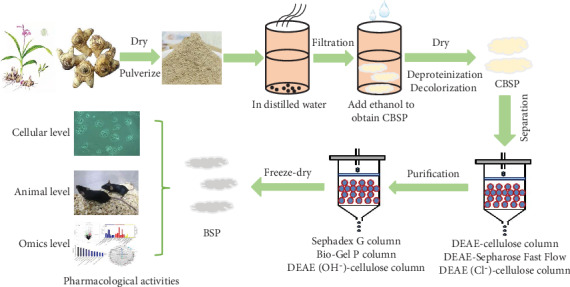
Schematic representation of the extraction, purification, and bioactivity of polysaccharides from *Bletilla striata*.

In order to illuminate the composition, molecular mass, and basic chemical structure of BSPs, further purification of crude polysaccharides is needed. The main purification method is column chromatography with various adsorbents and eluents. In this process, fractions containing polysaccharide products are collected, dialyzed, concentrated, and lyophilized to receive pure polysaccharides [28, 29]. As reported before, ion exchange chromatography (DEAE-cellulose and DEAE-Sepharose Fast Flow) could separate neutral polysaccharides from acidic ones using a salt gradient for elution, whereas gel filtration (Sephadex G column and Bio-Gel P column) could separate polysaccharides according to the differences of their molecular weights (Figure 1) [9, 19, 30]. For instance, Wang et al. fractionated four different parts with a DEAE-cellulose column (3.0 × 55 cm), preequilibrated with distilled water and eluted with 0, 0.05, 0.2, and 1.0 M of NaCl at a flow rate of 1.5 mL/min. The collected fraction was further purified on a Sephadex G-200 column (1 cm × 100 cm) and eluted with distilled water (10 mL/tube). Sugar-positive parts were gathered, concentrated, and lyophilized to obtain two homogeneous polysaccharides, BSP-1 and BSP-2, with an average molecular weight of 83.54 kDa and 12.60 kDa, respectively [9].

The procedures used to separate and purify polysaccharides from *B. striata* are summarized as follows [2]. The tubers of *B. striata* were overdried, finely pulverized, and sieved through a 30-mesh sieve, and then the powders were homogenized and dispersed in hot double-distilled water, then filtered to remove impurities. The crude extracts were precipitated by the addition of three vol. of 95% (*v*/*v*) ethanol and left to stand overnight. The resultant precipitate was collected by centrifugation. The last two steps were repeated to resuspend the precipitate in double-distilled water. Deproteinization was done by adding 1/3 vol. Sevage reagent (chloroform/n-butanol, 4 : 1) to the solution and stirring overnight, until the absorption of the solution at 260 nm and 280 nm was zero, then the aqueous phase was collected by centrifugation at 3500 rpm for 10 min. After decolorization, dialysis, evaporation, precipitation with ethanol, filtration, and lyophilization, the crude *B. striata* polysaccharides are obtained [2, 3, 5]. The crude extracts are redissolved and applied to different chromatographic columns described above, eluted with appropriate running buffers, collected, dialyzed, concentrated, and lyophilized, to produce the pure *B. striata* polysaccharide (BSP) [2]. Then, the polysaccharide contents are determined using the phenol-sulfuric acid method [31], and proteins in the polysaccharides are quantified with the Bradford method [32].

## 3. Physiochemical and Structural Features

The structural characteristics of plant polysaccharides, including molecular weight; monosaccharide composition and sequence; and types, positions, and configurations of glycosidic linkages as well, are the key to determine their unique physiochemical activity [21, 33, 34]. There are polysaccharides with various monosaccharide constituents and chemical structures isolated from *B. striata*. Meanwhile, the monosaccharide constituents and basic chemical structures of BSPs have been determined by several research groups through infrared spectroscopy (IR), nuclear magnetic resonance (NMR), gas chromatography-mass spectroscopy (GC-MS), gas chromatography (GC), high-performance liquid chromatography (HPLC), acid hydrolysis, methylation analysis, periodate oxidation, and Smith's degradation [7, 9, 18, 35]. The primary structural characteristics of BSPs and also their biological activities are summarized in Table 2, together with their related bibliographies.

### 3.1. Average Molecular Weights

HPLC and high-performance gel permeation chromatography (HPGPC) are mainly used to determine the average molecular weights of BSPs [8, 12]. Wang demonstrated that molecular weights of BSPs from different isolating fractions were 355.8 kDa (BSPS-I), 174.3 kDa (BSPS-II), 52.8 kDa (BSPS-III), 174.0 kDa (BSPS-IV), and 59.9 kDa (BSPS-V) [36]. It should be noticed that different experimental conditions may have impacts on the molecular weights of polysaccharides. Reports about the Mw of various *B. striata* polysaccharides ranged from 10^4^ to 10^5^ Da.

### 3.2. Monosaccharide Compositions

In general, with a process of acid-mediated cleavage of glycosidic linkages, derivatization, detection, and quantification with GC and HPLC, people could get the information of monosaccharide compositions [9, 14, 36]. And owing to different raw materials and purification processes, different monosaccharide compositions of BSPs are derived.

It is reported that most of BSPs are composed of mannose and glucose with a similar molar ratio [2, 14, 37]. Wang et al. separated two polysaccharides, BSP-1 and BSP-2, from *B. striata* and analyzed their monosaccharide compositions with GC [9]. The results are shown in Table 2. Diverse BSPs might have similar monosaccharide compositions in various molar ratios.

### 3.3. Chemical Structures

**Figure 2:**
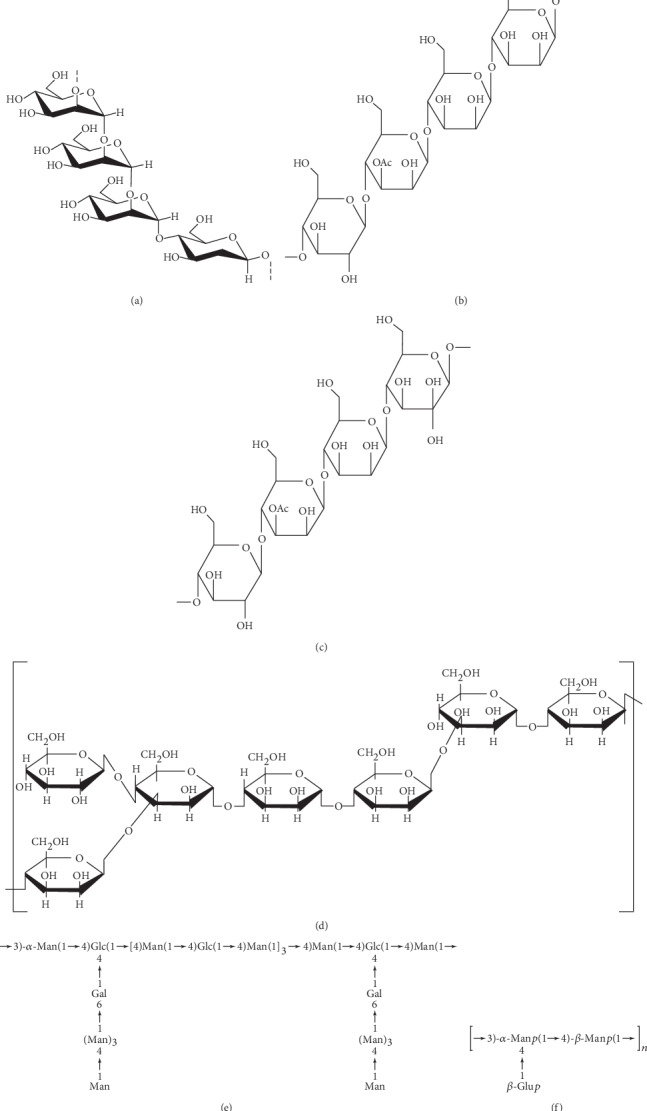
Schematic structures of *Bletilla striata* polysaccharides. (a) *Bletilla striata polysaccharide* b (BSPb) [15]. (b) Polysaccharide from the tuber of *Bletilla striata* (BSP-1) [9]. (c) Polysaccharide from the tuber of *Bletilla striata* (BSP-2) [9]. (d) Polysaccharide of *Bletilla striata* (BSP) [19]. (e) polysaccharide from the tuber of *Bletilla striata* (BSPF2) [14]. (f) *Bletilla striata* polysaccharide [37].

Besides monosaccharide components and molecular weights, information about structure or conformation of BSPs has been demonstrated. So far, all available evidence (Table 2) indicated that BSPs were a sort of glucomannan [2, 5]. An early publication in 1993 first reported that BSPs contained glucose and mannose in a molar ratio of 1 : 3, with the backbone consisting of 1,4-linked aldohexopyranosyl residues [37]. In addition, the structural features of a water-soluble polysaccharide (BSP-2) were studied by methylation analysis, GC-MS analysis, Fourier transform-infrared spectroscopy, ^1^H, ^13^C, HSQC, COSY, and HMBC NMR spectroscopy [9]. It was shown that the primarily BSP-2 structure was deduced to be →4)-*β*-D-Glc*p* (1→4)-*β*-D-Man*p* (1→4)-*β*-D-Man*p* (1→4)-*β*-D-Man*p*(1→. A schematic structure is shown in Figure 2(c). The primary structures of the *B. striata* polysaccharide (designated BSPF2), with a molecular weight of 2.35 × 10^5^ Da, containing mannose, glucose, and galactose in a molar ratio of 9.4 : 2.6 : 1.0, were determined with a combination of chemical and instrumental analyses, including methylation analysis, GC, methylation, and FT-IR. The backbone of BSPF2 consisted of (1→4)-linked mannosyl residues and (1→4)-linked glucosyl residues in a molar ratio of 2 : 1 [14].

As illustrated in Figure 2, the hypothesized structure of BSP fractions consists of two types, the linear chain and the branched chain, respectively, and the branched polysaccharides composed of mannose and glucose with different ratios are common [2].

### 3.4. Conformational Features

The activities of polysaccharides have a close relationship with their molecular weights, chemical structures, and chain conformations as well [21, 38]. However, few reports are available on the solution properties or chain conformations of BSPs. As for the advanced structure of BSPs, Qu et al. discovered that a shift in the maximum absorption wavelength (503-512 nm) of BSPs that appeared in distilled water was longer than that examined in the Congo red solution without BSPs (487-498 nm), indicating that BSPs exhibited a triple helical conformation [20]. The surfaces of BSP fractions exhibited a loose filamentous network, although there were large differences depending on concentration. Besides, some angular irregular voids were observed in the network structure. These voids possibly represent spaces that remained after ice crystals sublimated during the freeze-drying process [8].

The *B. striata* polysaccharide (BSP) could be used for preparing a fiber with mechanical properties through the environment-friendly method of phase inversion with ethanol as the nonsolvent. The prepared BSP fibers were woven into a fabric with excellent flexibility and had excellent flexibility with potential application in the curing of skin wounds [39]. This BSP hydrogel represented a preferable swelling ability and an appropriate water vapor transmission rate, and has been proven to control the inflammatory responses and accelerate the wound closure and thus has potential application in wound healing [40].

It is difficult to determine the definite relationship of the chain conformations, solution properties of BSPs, and their biological activities [41]. Further confirmation of the chain conformations of BSPs in an aqueous solution should require other investigations with advanced techniques, such as viscosity analyses, static and dynamic light scattering, circular dichroism, transmission electron microscopy, scanning electron microscopy, and atomic force microscopy in future research [21, 42].

## 4. Biological Activities

Based on the theory of traditional Chinese medicine, *B. striata* is widely used to treat alimentary canal mucosal damage, ulcers, bleeding, bruises, and burns [2, 36, 43]. In recent years, owing to their various biological properties and pharmacological functions, plant polysaccharides attract extensive attention in the field of biology and medicine. Similarly, more and more studies indicated that polysaccharides were a major class of bioactive compounds in *B. striata*, both in its beneficial effects on human health and its pharmacological value [2, 44, 45]. The multiple bioactivities and health benefits of BSPs are summarized and compared in detail below.

### 4.1. Immunomodulatory Activity

Working as immunomodulators or biological response modifiers, natural polysaccharides play a role in immunomodulation, which is considered to be an important biological function [46, 47]. The immunomodulatory activities of plant polysaccharides could be correlated with their structures, including molecular weights, chemical compositions, and glycosidic linkages [21, 48, 49]. Peng et al. reported that the *B. striata* polysaccharide fraction (BSPF2) could concentration-dependently induce spleen cell proliferation, and it was characterized by a strong stimulation of proliferation of mouse spleen cells [14].

The immunomodulatory activities of *B. striata* polysaccharides (BSP-1 and BSP-2) were previously investigated in rats treated with cyclophosphamide by Wang et al. [9]. Their results indicated that the thymus index inhibition rates of BSP-1 and BSP-2 were -23.5% and 3.3%, and the spleen index inhibition rates of mice with immune dysfunction were also increased, with inhibition rates of -36.0% and -24.4%, respectively. Furthermore, BSP-1 could significantly improve the immunological function of mice that had been immunosuppressed by cyclophosphamide and increase the thymus index in *in vivo* experiments [9, 24].

### 4.2. Antioxidant Activity

To study the mechanisms of traditional Chinese medicines as potential nutraceutical and therapeutic agents, investigating antioxidant activities is the focus of many researches, with various assay methods and activity indices [50, 51]. It has been reported that a wide range of biofunctional components of plants, fungi, and animals have strong antioxidant activities, among which polysaccharide is quite a representative one [52, 53].

Some research groups have demonstrated the antioxidative activities of *B. striata* polysaccharides *in vitro*. For example, Qu et al. recently demonstrated that BSPs have a definite antioxidative activity, estimated in 2,2-diphenyl-1-picrylhydrazyl (DPPH) radical, hydroxyl radical, and superoxide anion systems. BSPs display a dose-dependent DPPH radical scavenging effect of 42.20%, a hydroxyl radical scavenging effect of 35.97%, and a superoxide radical scavenging effect of 43.70% at the tested concentration of 5 mg/mL [20]. Besides, Cai et al. found that BSPs achieved by ultrasonic-microwave synergistic extraction had significantly higher hydroxyl radical and DPPH radical scavenging effects [54]. However, the *in vivo* antioxidant activities of BSPs are reported to be less marked.

### 4.3. Anti-Inflammatory Activity

It is reported that the anti-inflammatory effects of polysaccharides could be influenced by many factors, including their molecular size, form, degree of branching, and solubility in water [21, 53]. As we have mentioned before, many previous studies have suggested that polysaccharides exert strong anti-inflammatory activity through different mechanisms [53, 55]. The study reported by Yue et al. demonstrated that BSPb exhibited significant anti-inflammatory and antioxidative activities in Ang II-induced HMCs *via* the NOX4 and TLR2/MyD88 pathways, efficiently mediating the expression of key factors, such as NOX4 and TLR2, to attenuate the generation of ROS and inflammatory cytokines [12]. Lai et al. previously reported that BSPs could significantly downregulate inflammatory markers, *viz.*, L-1*β*, TNF-*α*, COX-2, and iNOS, and upregulate proinflammatory cytokines, like TIMP-1 and TGF-*β*1, and the anti-inflammatory properties of BSPs might be involved with the regulation of oxidative stress [18].

### 4.4. Antifibrotic Activity

Only few studies have demonstrated the direct antifibrotic effects of *B. striata* polysaccharides. More detailed studies are required to clarify the compositional features and antifibrotic activities of BSPs. Wang et al. reported that BSPb, isolated from the roots of *B. striata*, showed a dose-dependent effect on the proliferation of human mesangial cells (HMCs) and played an important role in protecting against the renal fibrosis effect, which is probably mediated by downregulating TGF*-β* RI, TGF-*β* RII, and *α*-SMA *in vitro* [15].

### 4.5. Hemostatic Activity

Pharmacological studies of plant polysaccharides have shed some light on a novel aspect of drug delivery in treating bleeding, bruises, and burns [2, 56]. Polysaccharides could promote tissue repair through different growth factors and produce healing effects by inducing endothelial cell proliferation and vascular endothelial growth factor expression or proinflammatory cytokine expression in macrophages [57, 58].

The research of Wang et al. previously reported that *B. striata* polysaccharides could upregulate vascular endothelial growth factor expression and enhance vascular endothelial cell (EC) proliferation *in vitro* in 2006 [37], and two years later they elucidated that the wound healing mechanism might be that the BSPs could induce coordinate changes in the mRNA levels of inducible nitric oxide synthase (iNOS), tumor necrosis factor alpha (TNF-*α*), and interleukin 1 beta (IL-1*β*) and then enhance the expression of these cytokines, but that the BSPs have no effect on interferon gamma (IFN-*γ*) level [59]. BSPs enhanced the proliferation and migration of L929 cells and significantly accelerated the wound healing process *in vivo* in full-thickness skin defect wounded models. The BSP hydrogel could accelerate wound healing and promote reepithelialization and collagen deposition by means of TGF-*β*/Smad signal pathway activation [60].

### 4.6. Gastrointestinal-Protective Effect

*B. striata* polysaccharides showed bioactivities in protecting the gastrointestinal mucosa. Such polysaccharides could promote tissue repair through different growth factors and produce anti-inflammatory effects by suppressing the neutrophil/cytokine cascade in intestinal epithelial organization. Luo et al. previously reported that BSPs alleviated intestinal epithelial barrier disruption in rats with thioacetamide- (TAA-) induced liver cirrhosis. They found that BSPs markedly reduced endotoxin levels, inhibited the inflammatory cytokines IL-6 and TNF-*α*, elevated expression of zonula occludens- (ZO-) 1 and occludin at tight junctions, and thereafter, improved the intercellular tight junctions [61, 62]. BSPs had the capacity to protect IEC-18 cells from LPS-induced injury, and the mechanisms could be associated with decreasing the inflammatory cytokine levels of IL-6 and TNF-*α* and elevating the expression of ZO-1 and occludin, which might serve as a new protective agent for LPS-induced intestinal epithelial barrier disruption [63]. The gastroprotective mechanisms of BSP could be related to the mitigation of oxidative stress, neutrophil infiltration, and inflammatory cytokine accumulation. The inhibition of MAPK/NF-*κ*B signaling pathway activation was mediated by BSP, and pretreatment with BSP promoted the production of acid mucus and upregulated endogenous PGE 2 production, which protected the gastric mucosa from damage induced by ethanol [64].

### 4.7. Other Biological Activities

*B. striata* polysaccharides were shown to have significant *in vivo* and *in vitro* antitumor activities [2]. Li and Bai previously investigated the *in vivo* and *in vitro* effects of paclitaxel nanoparticle- (PTX-) loaded BSPs on human gastric cancer cells. The results suggested that BSP-loaded paclitaxel nanoparticles could realize enhanced drug delivery and exert an antiproliferative effect on the human gastric gland cancer cell line (MKN45) effectively and safely both *in vivo* and *in vitro* [10].

The lifespan of *C. elegans* was extended, and its locomotion ability and stress resistance were increased and mRNA levels of *age-1* and *hcf-1* were reduced after BSP treatment. BSP could produce an antiaging effect on *C. elegans* through the insulin/IGF signaling pathway [65].

## 5. Conclusion and Future Perspectives

As a classical traditional Chinese medicine, *B. striata* has been extensively investigated in the past decades. Among the various active ingredients of this plant, polysaccharides attract broad attention by virtue of their excellent biological properties and pharmacological functions. Early research has been done and published on the extraction, purification, and preliminary structural identification as well as on the biological functions of *B. striata* polysaccharides. In this paper, we reviewed recent advances in research on the structure and bioactivities of polysaccharides from *B. striata*.

Over the past decades, phytochemical studies have isolated a variety of *B. striata* polysaccharides. The structural diversity and heterogeneity make research challenging in terms of structural perspectives.

Several reports have systematically explored the chemical structures of BSPs. The main methods used for characterization in these studies were still HPGPC, partial acid, oxidation with periodic acid, Smith's degradation, HPLC, GC, IR, ^1^H-^13^C-NMR, and GC-MS. It is reported that the glycan backbone of BSPs is mainly represented by 1,4-linked mannosyl residues and 1,4-linked glucosyl residues, with few side chains attached to *O*‐2, *O*‐3, or *O*‐6, accompanied by different branching and terminal sites.

Besides, several studies have focused on the pharmacological functions of *B. striata* polysaccharides, *e.g.*, antioxidant, antifibrotic, and hemostatic activities, while few studies focused on the relationship between chemical structure and biological activity. Further investigations are required to fully unfold these mysteries. Similarly, more preclinical and clinical trials are required to confirm the reliability and effectiveness of *B. striata* polysaccharides in human health, as *in vitro* observations cannot accurately reflect *in vivo* effects.

On the other hand, the development of new “omic” technologies, such as microbiomics, transcriptomics, metabolomics, and proteomics, and current techniques including genomics and bioinformatics will definitely contribute to uncover the mechanisms of various biological activities of *B. striata* polysaccharides.

As more attention is given and new research methods have arisen, the significant medicinal value of *Bletilla striata* polysaccharides will be featured, and their wide exploitation and utilization in the field of biomedicine and functional food can be expected in the near future.

## Figures and Tables

**Table 1:** A summary of the extraction of polysaccharides from *Bletilla striata.*

No.	Times (min)	Solid-liquid ratio	Temperature (°C)	Other conditions	Yield (%)	References
*Routine extraction*						
1	150	1 : 30	80	2 times		[18]
2	180	1 : 10	60		18.50	[24]
3	240	1 : 5	80	1 time		[66]
4	180	1 : 15	90	Cold soaking extraction 6 h, first 2 times	28.43	[67]
5	90	1 : 30	80		26.45	[43]
6	180	1 : 10	90	3 times	10.00	[68]
7	90	1 : 10	90	2 times	7.64	[69]
8	180	1 : 6	90	Alkali solution, 3 times		[70]
9	240		95	0.2 M NaOH, 3 times	62.50	[36]
*Ultrasound-assisted extraction*						
1	50	1 : 50	60	400 W, 2 times	26.023	[25]
2	5	1 : 20		50 W	3.23	[54]
3	20	1 : 25		100 W	12.343	[71]
4	80	1 : 30	30	126 W	19.94	[72]
*Microwave-assisted extraction*						
1	5	1 : 63		504 W, pH 7.0	32.48	[26]
2	5	1 : 20		200 W	6.14	[54]
3	5	1 : 20		Ultrasonic 50 W Microwave 200 W	6.98	[54]
*Infrared-assisted extraction*						
1	150	1 : 53	75	1 time	43.95	[20]

**Table 2:** The polysaccharides isolated from *Bletilla striata.*

No.	Compound name	Molecular weight	Monosaccharide composition	Structures	Biological activities	Reference
1	BSP-1	8.354 × 10^4^ Da	Man and Glc in the ratio of 4.0 : 1.0	Backbone composed of *β*-1,4-linked Man*p*	Immunomodulation	[9]
	BSP-2	1.26 × 10^4^ Da	Man and Glc in the ratio of 3.0 : 1.0	Backbone composed of *β*-1,4-linked Glc*p*		[9]
2	BSP	Mw : 3.73 × 10^5^ g/mol Mn : 6.75 × 10^4^ g/mol	Man and Glc in the ratio of 2.946 : 1	Backbone composed of 1,4-linked Glc*p*. Branches composed of 1,3-linked Man*p* and 1,3-linked Glc*p*	Anti-inflammatory	[7]
3	BSP	1.98 × 10^5^ Da		*β*-Glucopyranose and *α*-mannopyranose	Antitumor	[18]
4	BFPS	Mw : 9.545 × 10^4^ g/mol Mn : 7.297 × 10^4^ g/mol	Man and Glc in the ratio of 7.4 : 2.6			[8]
	BVPS	Mw : 1.472 × 10^5^ g/mol Mn : 1.218 × 10^5^ g/mol	Man and Glc in the ratio of 7.37 : 2.63			[8]
5	BSPF2	2.35 × 10^5^ Da	Man, Glc, and Gal in the ratio of 9.4 : 2.6 : 1.0	Backbone composed of 1,4-linked Man*p* and 1,4-linked Glc*p* in a molar ratio of 2 : 1	Immunomodulation	[14]
6	BSP		Man and Glc in the ratio of 1.616 : 1.962	Backbone composed of 1,3,4-linked Man*p* and 1-linked Man*p*. Branches composed of 1-linked Glc*p*		[37]
7	BSPb	2.60 × 10^5^ Da	Glc and Man in the ratio of 3 : 1	Backbone composed of 1,2-linked Man*p* and 1,4-linked Glc*p*	Antifibrotic effect Anti-inflammatory	[12, 15]
8	BSP	1.82 × 10^5^ Da	Man and Glc in the ratio of 3 : 1	1,4-Linked aldohexopyranosyl residues		[24]
9	BOP	4.93 × 10^5^ Da	Man and Glc in the ratio of 7.88 : 2.12	Backbone composed of 1,4-linked Man*p* and 1,4-linked Glc*p* in a molar ratio of 4 : 1	Immunomodulation Antitumor	[43]
10	BSP	~1.35 × 10^5^ Da	Man and Glc in the ratio of 2.4 : 1	Backbone composed of 1,4-linked Man*p*	Anti-inflammatory	[37, 73]
11	BSPI-A	>4.0 × 10^5^ Da	Man and Glc in the ratio of 8.09 : 1	Backbone composed of 1,4-linked Man*p* and 1,4-linked Glc*p*		[74]
12	BT		Man and Glc in the ratio of 2.88 : 1	Backbone composed of 1,4-linked Man*p* and 1,4-linked Glc*p*		[75]
13	BSP	~1.0 × 10^5^ Da	Man and Glc in the ratio of 1.6 : 1		Cell proliferation	[76]
14	BSP	~2.0 × 10^5^ Da	Man and Glc in the ratio of 3.76 : 1	Backbone composed of 1,4-linked Man*p* and 1,4-linked Glc*p*		[77]
15	BSP		Man and Glc	Backbone composed of 1,4-linked Man*p* and 1,4-linked Glc*p*	Cell proliferation	[17]
16	pFSP	9.1 × 10^4^ Da	Man, Glc, and Gal in the ratio of 3.45 : 1.00 : 2.03	Backbone composed of (1→4)-linked-*α*-D-Glc*p*, (1→4)-linked-*β*-D-Man*p*, and (1→3,6)-linked-*β*-D-Man*p*. Branches composed of (1→6)-linked-*β*-D-Gal*p* and terminated with (1→)-linked-*β*-D-Man*p*	Antioxidant	[78]
17	BSP	1.46 × 10^5^ Da	Man and Glc in the ratio of 2.4 : 1.0	*α*-Man and *β*-Glc residues	Anti-inflammatory	[79]

## References

[1] He X., Wang X., Fang J. (2017). *Bletilla striata*: medicinal uses, phytochemistry and pharmacological activities. *Journal of Ethnopharmacology*.

[2] Chen Z., Cheng L., He Y., Wei X. (2018). Extraction, characterization, utilization as wound dressing and drug delivery of *Bletilla striata* polysaccharide: a review. *International Journal of Biological Macromolecules*.

[3] Zhao Y., Wang Y. (2015). Research progress of polysaccharides from *Bletilla striata*. *Traditional Medical Science and Technology*.

[4] Zhang M., Shao Q., Xu E., Wang Z., Wang Z., Yin L. (2019). *Bletilla striata*: a review of seedling propagation and cultivation modes. *Physiology and Molecular Biology of Plants*.

[5] Chen J., Lv L., Li Y. (2019). Preparation and evaluation of *Bletilla striata* polysaccharide/graphene oxide composite hemostatic sponge. *International Journal of Biological Macromolecules*.

[6] Cai J., Liang Y., Wu Q., Hu J., Liang H., Wei K. (2018). Optimization of extraction of polysaccharide from *Bletilla striata* and its biological activity. *Food industry*.

[7] Liao Z., Zeng R., Hu L., Maffucci K. G., Qu Y. (2019). Polysaccharides from tubers of *Bletilla striata*: physicochemical characterization, formulation of buccoadhesive wafers and preliminary study on treating oral ulcer. *International Journal of Biological Macromolecules*.

[8] Kong L., Yu L., Feng T., Yin X., Liu T., Dong L. (2015). Physicochemical characterization of the polysaccharide from *Bletilla striata*: effect of drying method. *Carbohydrate Polymers*.

[9] Wang Y., Han S., Li R. (2019). Structural characterization and immunological activity of polysaccharides from the tuber of *Bletilla striata*. *International Journal of Biological Macromolecules*.

[10] Xuchen L., Guang B. (2019). *In vivo* and *in vitro* effects of *Bletilla striata* polysaccharide-loaded paclitaxel nanoparticles on human gastric cancer cells. *Tropical Journal of Pharmaceutical Research*.

[11] Wang L., Wu Y., Li J., Qiao H., Di L. (2018). Rheological and mucoadhesive properties of polysaccharide from *Bletilla striata* with potential use in pharmaceutics as bio-adhesive excipient. *International Journal of Biological Macromolecules*.

[12] Yue L., Wang W., Wang Y. (2016). *Bletilla striata* polysaccharide inhibits angiotensin II-induced ROS and inflammation via NOX4 and TLR2 pathways. *International Journal of Biological Macromolecules*.

[13] Zhan X., Jia L., Niu Y. (2014). Targeted depletion of tumour-associated macrophages by an alendronate-glucomannan conjugate for cancer immunotherapy. *Biomaterials*.

[14] Peng Q., Li M., Xue F., Liu H. (2014). Structure and immunobiological activity of a new polysaccharide from *Bletilla striata*. *Carbohydrate Polymers*.

[15] Wang Y., Liu D., Chen S., Wang Y., Jiang H., Yin H. (2014). A new glucomannan from *Bletilla striata*: structural and anti-fibrosis effects. *Fitoterapia*.

[16] Hu L., Liao Z., Hu Q., Maffucci K. G., Qu Y. (2018). Novel *Bletilla striata* polysaccharide microneedles: fabrication, characterization, and *in vitro* transcutaneous drug delivery. *International Journal of Biological Macromolecules*.

[17] Wang C., Luo W., Li P. (2017). Preparation and evaluation of chitosan/alginate porous microspheres/*Bletilla striata* polysaccharide composite hemostatic sponges. *Carbohydrate Polymers*.

[18] Lai Y. L., Lin Y. Y., Sadhasivam S. (2018). Efficacy of *Bletilla striata* polysaccharide on hydrogen peroxide-induced apoptosis of osteoarthritic chondrocytes. *Journal of Polymer Research*.

[19] Cui X., Zhang X., Yang Y., Wang C., Zhang C., Peng G. (2016). Preparation and evaluation of novel hydrogel based on polysaccharide isolated from *Bletilla striata*. *Pharmaceutical Development and Technology*.

[20] Qu Y., Li C., Zhang C., Zeng R., Fu C. (2016). Optimization of infrared-assisted extraction of *Bletilla striata* polysaccharides based on response surface methodology and their antioxidant activities. *Carbohydrate Polymers*.

[21] Ji X., Peng Q., Yuan Y., Shen J., Xie X., Wang M. (2017). Isolation, structures and bioactivities of the polysaccharides from jujube fruit (*Ziziphus jujuba* Mill.): a review. *Food Chemistry*.

[22] Ji X., Han L., Liu F., Yin S., Peng Q., Wang M. (2019). A mini-review of isolation, chemical properties and bioactivities of polysaccharides from buckwheat (*Fagopyrum Mill*). *International Journal of Biological Macromolecules*.

[23] Shang J. (2018). Progress on extraction of polysaccharide from *Bletilla striata* and its application in food. *Modern Food*.

[24] Wang Y. (2019). *Isolation, characterization and immunological activity of polysaccharides from the tuber of Bletilla striata (M.S. thesis)*.

[25] He X., Gu F., Huang S., Han B., Chen N. (2017). Ultrasonic-assisted extraction and *in vitro* antioxidant activities evaluation of polysaccharides from *Bletilla striata*. *Western Anhui University*.

[26] Han W., Peng R., Zhao S. (2018). Extraction optimization of total polysaccharide from *Bletilla striata* by response surface methodology with Plackett-Burman design. *Journal of Xuzhou Institute of Technology*.

[27] Tai R., Yin X. (2014). Extraction and analysis of polysaccharide from *Bletilla Striata*. *China Pharmaceutical*.

[28] Zhang J., Wen C., Duan Y., Zhang H., Ma H. (2019). Advance in *Cordyceps militaris* (Linn) link polysaccharides: isolation, structure, and bioactivities: a review. *International Journal of Biological Macromolecules*.

[29] Ji X., Hou C., Guo X. (2019). Physicochemical properties, structures, bioactivities and future prospective for polysaccharides from *Plantago* L. (*Plantaginaceae*): a review. *International Journal of Biological Macromolecules*.

[30] Zhu J., Guo X., Guo T. (2018). Novel pH-responsive and self-assembled nanoparticles based on *Bletilla striata* polysaccharide: preparation and characterization. *RSC Advances*.

[31] DuBois M., Gilles K. A., Hamilton J. K., Rebers P. A., Smith F. (1956). Colorimetric method for determination of sugars and related substances. *Analytical Chemistry*.

[32] Bradford M. M. (1976). A rapid and sensitive method for the quantitation of microgram quantities of protein utilizing the principle of protein-dye binding. *Analytical Biochemistry*.

[33] Masci A., Carradori S., Casadei M. A. (2018). *Lycium barbarum* polysaccharides: extraction, purification, structural characterisation and evidence about hypoglycaemic and hypolipidaemic effects. A review. *Food Chemistry*.

[34] Li Y., Xu F., Zheng M., Xi X., Cui X., Han C. (2018). Maca polysaccharides: a review of compositions, isolation, therapeutics and prospects. *International Journal of Biological Macromolecules*.

[35] Guan Q., Zhang G., Sun D. (2017). *In vitro* and *in vivo* evaluation of docetaxel-loaded stearic acid-modified *Bletilla striata* polysaccharide copolymer micelles. *PloS One*.

[36] Wang W. (2015). *Physicochemical properties and pharmacological activities of polysaccharides alkali extracted from Bletilla striata (M.S. thesis)*.

[37] Wang C., Sun J., Luo Y. (2006). A polysaccharide isolated from the medicinal herb *Bletilla striata* induces endothelial cells proliferation and vascular endothelial growth factor expression *in vitro*. *Biotechnology Letters*.

[38] Nie S. P., Xie M. Y. (2011). A review on the isolation and structure of tea polysaccharides and their bioactivities. *Food Hydrocolloids*.

[39] Zhuang Y., Wang L., Liu C. (2019). A novel fiber from *Bletilla striata* tuber: physical properties and application. *Cellulose*.

[40] Luo Y., Diao H., Xia S., Dong L., Chen J., Zhang J. (2010). A physiologically active polysaccharide hydrogel promotes wound healing. *Journal of Biomedical Materials Research Part A*.

[41] Yan J. K., Wang W. Q., Wu J. Y. (2014). Recent advances in *Cordyceps sinensis* polysaccharides: mycelial fermentation, isolation, structure, and bioactivities: a review. *Journal of Functional Foods*.

[42] Yang L., Zhang L. M. (2009). Chemical structural and chain conformational characterization of some bioactive polysaccharides isolated from natural sources. *Carbohydrate Polymers*.

[43] Wang S. (2017). *Structural analysis and antitumor activity of polysaccharides from Bletilla Ochracea Schltr (M.S. thesis)*.

[44] Lv H., Zhang T., Li Q. (2015). Advances in pharmacological action of polysaccharides from *Bletilla striata*. *China Pharm*.

[45] Qu Y., Zhang C., Liao Z., Hu L., He Y. (2017). Exploration on pharmaceutical applications of *Bletilla striata* polysaccharide in medical biomaterials. *Pharmacy and Clinics of Chinese Materia Medica*.

[46] Ramberg J. E., Nelson E. D., Sinnott R. A. (2010). Immunomodulatory dietary polysaccharides: a systematic review of the literature. *Nutrition Journal*.

[47] Rahbar Saadat Y., Yari Khosroushahi A., Pourghassem Gargari B. (2019). A comprehensive review of anticancer, immunomodulatory and health beneficial effects of the lactic acid bacteria exopolysaccharides. *Carbohydrate Polymers*.

[48] Liu J., Bai R., Liu Y., Zhang X., Kan J., Jin C. (2018). Isolation, structural characterization and bioactivities of naturally occurring polysaccharide-polyphenolic conjugates from medicinal plants—a review. *International Journal of Biological Macromolecules*.

[49] Ji X., Shen Y., Guo X. (2018). Isolation, structures, and bioactivities of the polysaccharides from *Gynostemma pentaphyllum* (Thunb.) Makino: a review. *BioMed Research International*.

[50] Wang J., Hu S., Nie S., Yu Q., Xie M. (2016). Reviews on mechanisms of *in vitro* antioxidant activity of polysaccharides. *Oxidative Medicine and Cellular Longevity*.

[51] Wang Z. J., Xie J. H., Nie S. P., Xie M. Y. (2017). Review on cell models to evaluate the potential antioxidant activity of polysaccharides. *Food & Function*.

[52] Alam M., Bristi N., Rafiquzzaman M. (2013). Review on *in vivo* and *in vitro* methods evaluation of antioxidant activity. *Saudi Pharmaceutical Journal*.

[53] Zhu F., Du B., Xu B. (2017). Anti-inflammatory effects of phytochemicals from fruits, vegetables, and food legumes: a review. *Critical Reviews in Food Science and Nutrition*.

[54] Cai J., Xiong J., Huang Y. (2016). Study on ultrasonic-microwave synergistic extraction of polysaccharose from *Bletilla striata* and its antioxidant activity. *Food Industry Science & Technology*.

[55] Muszynska B., Grzywacz-Kisielewska A., Kala K., Gdula-Argasinska J. (2018). Anti-inflammatory properties of edible mushrooms: a review. *Food Chemistry*.

[56] Wang Y., Liu J., Li Q., Wang Y., Wang C. (2015). Two natural glucomannan polymers, from *Konjac* and *Bletilla*, as bioactive materials for pharmaceutical applications. *Biotechnology Letters*.

[57] Yang X., Liu W., Li N. (2017). Design and development of polysaccharide hemostatic materials and their hemostatic mechanism. *Biomaterials Science*.

[58] Cui X., Wang S., Cao H. (2018). A review. The bioactivities and pharmacological applications of *Polygonatum sibiricum* polysaccharides. *Molecules*.

[59] Diao H., Li X., Chen J. (2008). *Bletilla striata* polysaccharide stimulates inducible nitric oxide synthase and proinflammatory cytokine expression in macrophages. *Journal of Bioscience and Bioengineering*.

[60] Zhang C., He Y., Chen Z., Shi J., Qu Y., Zhang J. (2019). Effect of polysaccharides from *Bletilla striata* on the healing of dermal wounds in mice. *Evidence-Based Complementary and Alternative Medicine*.

[61] Luo L., Zhou Z., Xue J. (2018). *Bletilla striata* polysaccharide has a protective effect on intestinal epithelial barrier disruption in TAA-induced cirrhotic rats. *Experimental and Therapeutic Medicine*.

[62] Zhang H. (2018). *Protective effect of polysaccharide of Bletilla striata on ethanol-induced gastric mucosal injury (M.S. thesis)*.

[63] Yang F., Luo L., Liu Y. (2019). *Bletilla striata* polysaccharides ameliorates lipopolysaccharide-induced injury in intestinal epithelial cells. *Saudi Journal of Gastroenterology*.

[64] Liu Q., Wei X., Tang Y. (2014). To investigate the effect of *Rhizoma Bletillae* polysaccharide and its combination with 5-Fu on human gastric cancer cell line MKN45. *Inner Mongol Journal of Traditional Chinese Medicine*.

[65] Zhang Y. S., Lv T., Li M. (2015). Anti-aging effect of polysaccharide from *Bletilla striata* on nematode *Caenorhabditis elegans*. *Pharmacognosy Magazine*.

[66] Zhang Q., Wang N., Hu R. (2015). Wet spinning of *Bletilla striata* polysaccharide/silk fibroin hybrid fibers. *Materials Letters*.

[67] Li Q., Li K., Huang S. S., Zhang H. L., Diao Y. P. (2014). Optimization of extraction process and antibacterial activity of *Bletilla striata* polysaccharides. *Asian Journal of Chemistry*.

[68] Han L., Fu Y., Shen X., Zhang L. (2008). Optimization of the extraction technology of polysaccharide from *Rhizoma Bletiliae*. *China Pharm*.

[69] Zhao N., Li Z., Zhang Q., Zhang C. (2015). Study on extraction technology of *Bletilla striata* polysaccharide. *Applied Chemical Industry*.

[70] Peng R., Zhao S., Wang C., Han W. (2019). Optimization of flocculating purification on *Bletilla striata* polysaecharide by desirability function and response surface methodology combined with Plackett-Burman test. *Journal of Nanjing Tech University*.

[71] Xiang Y., Ye Q., Li W., Xu W., Yang H. (2014). Preparation of wet-spun polysaccharide fibers from Chinese medicinal *Bletilla striata*. *Materials Letters*.

[72] Liu Y., Han W. (2017). Ultrasonic-assisted extraction of polysaecharide from *Bleailla striata*. *Mechanical and Electrical Information*.

[73] Dong L., Xia S., Luo Y. (2009). Targeting delivery oligonucleotide into macrophages by cationic polysaccharide from *Bletilla striata* successfully inhibited the expression of TNF-*α*. *Journal of Controlled Release*.

[74] Wang B., Sha X., Huang L., Wang Z. (2010). Isolation, purification and structural characterization of a polysaccharide fraction from stem tuber of *Bletilla striata*, named BSPI-A. *Food Science*.

[75] Liu J., Wang H., Yin Y., Li N., Cai P., Yang S. (2012). Controlled acetylation of water-soluble glucomannan from *Bletilla striata*. *Carbohydrate Polymers*.

[76] Wu X.-g., Xin M., Chen H., Yang L.-n., Jiang H.-r. (2010). Novel mucoadhesive polysaccharide isolated from *Bletilla striata* improves the intraocular penetration and efficacy of levofloxacin in the topical treatment of experimental bacterial keratitis. *Journal of Pharmacy and Pharmacology*.

[77] Zhang M., Sun L., Zhao W. (2014). Cholesteryl-modification of a glucomannan from *Bletilla striata* and its hydrogel properties. *Molecules*.

[78] Chen Z., Zhao Y., Zhang M. (2020). Structural characterization and antioxidant activity of a new polysaccharide from *Bletilla striata* fibrous roots. *Carbohydrate Polymers*.

[79] Zhang C., Gao F., Gan S. (2019). Chemical characterization and gastroprotective effect of an isolated polysaccharide fraction from *Bletilla striata* against ethanol-induced acute gastric ulcer. *Food and Chemical Toxicology*.

